# Age Drives Distortion of Brain Metabolic, Vascular and Cognitive Functions, and the Gut Microbiome

**DOI:** 10.3389/fnagi.2017.00298

**Published:** 2017-09-25

**Authors:** Jared D. Hoffman, Ishita Parikh, Stefan J. Green, George Chlipala, Robert P. Mohney, Mignon Keaton, Bjoern Bauer, Anika M. S. Hartz, Ai-Ling Lin

**Affiliations:** ^1^Sanders-Brown Center on Aging, University of Kentucky Lexington, KY, United States; ^2^Depatment of Pharmacology and Nutritional Science, University of Kentucky Lexington, KY, United States; ^3^Research Resources Center, University of Illinois at Chicago Chicago, IL, United States; ^4^Metabolon Inc. Durham, NC, United States; ^5^Department of Pharmaceutical Sciences, University of Kentucky Lexington, KY, United States; ^6^Department of Engineering, University of Kentucky Lexington, KY, United States

**Keywords:** aging, MRI, brain metabolism, neurovascular function, gut microbiome, cognition, anxiety, Alzheimer’s disease

## Abstract

Advancing age is the top risk factor for the development of neurodegenerative disorders, including Alzheimer’s disease (AD). However, the contribution of aging processes to AD etiology remains unclear. Emerging evidence shows that reduced brain metabolic and vascular functions occur decades before the onset of cognitive impairments, and these reductions are highly associated with low-grade, chronic inflammation developed in the brain over time. Interestingly, recent findings suggest that the gut microbiota may also play a critical role in modulating immune responses in the brain via the brain-gut axis. In this study, our goal was to identify associations between deleterious changes in brain metabolism, cerebral blood flow (CBF), gut microbiome and cognition in aging, and potential implications for AD development. We conducted our study with a group of young mice (5–6 months of age) and compared those to old mice (18–20 months of age) by utilizing metabolic profiling, neuroimaging, gut microbiome analysis, behavioral assessments and biochemical assays. We found that compared to young mice, old mice had significantly increased levels of numerous amino acids and fatty acids that are highly associated with inflammation and AD biomarkers. In the gut microbiome analyses, we found that old mice had increased *Firmicutes*/*Bacteroidetes* ratio and alpha diversity. We also found impaired blood-brain barrier (BBB) function and reduced CBF as well as compromised learning and memory and increased anxiety, clinical symptoms often seen in AD patients, in old mice. Our study suggests that the aging process involves deleterious changes in brain metabolic, vascular and cognitive functions, and gut microbiome structure and diversity, all which may lead to inflammation and thus increase the risk for AD. Future studies conducting comprehensive and integrative characterization of brain aging, including crosstalk with peripheral systems and factors, will be necessary to define the mechanisms underlying the shift from normal aging to pathological processes in the etiology of AD.

## Introduction

Advancing age increases the risk factor for developing dementia, with imaging and biomarker data suggesting that the pathophysiological processes of Alzheimer’s disease (AD) begin more than a decade prior to the diagnosis of dementia (Reiman et al., 2004; Reitz et al., 2011; Bangen et al., 2013). However, how aging processes contribute to AD etiology still remains unclear. Bioenergetic imbalance over time has been considered as one of the primary causes for these chronic disorders (Wallace, 2011). In the central nervous system, brain energy supply declines with age (Petit-Taboué et al., 1998). Failure to maintain brain metabolism causes dysfunctional cellular energy status and nucleotide biosynthesis (Ivanisevic et al., 2016), leading to cognitive impairment and brain volume atrophy (Everson-Rose and Ryan, 2015). In addition, this energetic imbalance leads to neuroinflammation accompanied by reduced neuronal activity and increased glial activation (Boumezbeur et al., 2010; Lynch et al., 2010; O’Neill et al., 2016; Ron-Harel et al., 2016). Glial over-activation can cause release of inducible nitric oxide synthase (iNOS), which can result in inflammation and sepsis (Wong et al., 1996). Chronic neuroinflammation can further lead to retention of amyloid beta (Aβ) plaques and tau tangles as seen in AD, and ultimately, memory loss and dementia (Wyss-Coray and Rogers, 2012).

Emerging evidence shows the metabolic imbalance and inflammatory responses may not only originate in the brain *per se*, but also from gut microbiota (Kau et al., 2011). The gut microbiota is the community of microorganisms in the intestines that contains over 1000 different bacterial species, categorized into four primary phyla: *Firmicutes*, *Bacteroidetes*, *Actinobacteria* and *Proteobacteria* (Verbeke et al., 2014). A number of studies have demonstrated that the gut microbiota changes with age (Biagi et al., 2010; Claesson et al., 2011; Langille et al., 2014). In particular, an increased *Firmicutes/Bacteroidetes* (F/B) ratio is associated with increased inflammation and excess energy harvest from food in obese patients (Verdam et al., 2013). Increased F/B ratio is associated with the weakening of the epithelial tight junctions, allowing proinflammatory cytokines produced by pathogenic bacteria transfer to the brain from the blood stream or vagus nerve (Braniste et al., 2014; Al-Asmakh and Hedin, 2015). Inflammation due to leaky gut syndrome has also been shown to increase the risk for anxiety and depression (Dinan and Cryan, 2013), which can exacerbate learning and memory performance (Foster, 2016; Sharon et al., 2016).

Leaky gut syndrome also leads to neurovascular defects, evident by increased blood-brain barrier (BBB) permeability (Braniste et al., 2014). The reduction of BBB transporters may lead to impaired clearance of Aβ (Zlokovic, 2011; Bell et al., 2012), enhancing the risk of dementia like symptoms (Erickson and Banks, 2013). Impaired BBB function is also linked with reduced cerebral blood flow (CBF; Bell et al., 2012). Indeed, reductions in CBF with age have been known for years (Melamed et al., 1980). These neurovascular risks are highly associated with accelerated decline in language ability, verbal memory, attention and visuospatial abilities, and increased anxiety and depression in aging (Gur et al., 1987; Ebmeier et al., 1997; Zlokovic, 2011; Bangen et al., 2013; Park and Moghaddam, 2017).

Collectively, the cognitive aging and risk for AD may be driven by deleterious changes of brain physiology originated from the central nervous system as well as the peripheral systems. Nonetheless, the associations between brain metabolism, perfusion, cognition and gut microbiome remain largely unknown. In this study, our objective was to examine the effects of aging on the brain and the gut in young and old mice and how these effects collectively alter neurological function. To achieve this, we used metabolomics for brain metabolite assessment, 16s ribosomal RNA (rRNA) gene amplicon sequencing to analyze the gut microbiome, neuroimaging to examine brain vascular functions, and behavioral testing to determine memory and anxiety. We hypothesized that age-related deleterious changes would be exhibited in the brain and gut, effecting brain metabolic, vascular and cognitive functions, which may increase the risk for developing AD.

## Materials and Methods

### Animals

Young (5–6 months) and old (18–20 months) male C57BL/6N mice were acquired from the National Institute of Aging Colony. We determined the sample size via power analysis to ensure comparison at a 0.05 level of significance and 90% chance of detecting a true difference of each measured variable between the two groups. Each mouse was caged individually and housed in a specific pathogen-free facility. In order to avoid the potential for aggression when combining multiple male mice into one cage, mice were housed individually. Further, mice should be housed individually for gut microbiome analysis due to cage effects from microbiome transfer, e.g., mice eating each other’s feces giving them a very similar gut microbiome and thus, the mice would be *N* = 1 for that particular cage (Laukens et al., 2016). The mice were weighed weekly and given *ad libitum* access to food and water. All experimental procedures were performed according to NIH guidelines and approved by the Institutional Animal Care and Use Committee (IACUC) at the University of Kentucky (UK).

### Gut Microbiome Analysis

#### Fecal DNA Amplification

Fecal samples were collected from young (*N* = 39) and old (*N* = 28) mice and frozen at −80°C until further use. A PowerSoil DNA Isolation Kit (MO BIO Laboratories, Inc.) was used for fecal DNA extraction, according to the manufacturer’s protocol. Genomic DNA was PCR amplified with primers CS1_515F and CS2_926R (Walters et al., 2015) targeting the V4-V5 regions of microbial 16S rRNA genes using a two-stage “targeted amplicon sequencing (TAS)” protocol (Bybee et al., 2011; Green et al., 2015). First stage amplifications were performed with the following thermocycling conditions: 95°C for 3 min, 28 cycles of 95°C for 45 s, 55°C for 45 s, 72°C for 90 s and final elongation at 72°C for 10 min. Barcoding was performed using a second-stage PCR amplification with Access Array Barcode Library for Illumina Sequencers (Fluidigm, South San Francisco, CA, USA; Item# 100-4876). The pooled libraries, with a 15% phiX spike-in, were loaded on a MiSeq v3 flow cell, and sequenced using an Illumina MiSeq sequencer, with paired-end 300 base reads. Fluidigm sequencing primers, targeting the CS1 and CS2 linker regions, were used to initiate sequencing. De-multiplexing of reads was performed on instrument. Second stage PCR amplification and library pooling was performed at the DNA Services (DNAS) facility, Research Resources Center (RRC), University of Illinois at Chicago (UIC). Sequencing was performed at the W.M. Keck Center for Comparative and Functional Genomics at the University of Illinois at Urbana-Champaign (UIUC).

#### Microbial Analysis

Forward and reverse reads were merged using PEAR (Zhang et al., 2014). Primer sequences were identified using Smith-Watermann alignment and trimmed from the sequence. Reads that lacked either primer sequence were discarded. Sequences were then trimmed based on quality scores using a modified Mott algorithm with PHRED quality threshold of *p* = 0.01, and sequences shorter than 300 bases after trimming were discarded. QIIME v1.8 was used to generate operational taxonomic unit (OTU) tables and taxonomic summaries (Caporaso et al., 2010). Briefly, the resulting sequence files were merged with sample information. OTU clusters were generated in a *de novo* manner using the UCLUST algorithm with a 97% similarity threshold (Edgar, 2010). Chimeric sequences were identified using the USEARCH61 algorithm with the GreenGenes 13_8 reference sequences (McDonald et al., 2012). Taxonomic annotations for each OTU were using the UCLUST algorithm and GreenGenes 13_8 reference with a minimum similarity threshold of 90% (Edgar, 2010; McDonald et al., 2012). Taxonomic and OTU abundance data were merged into a single OTU table and summaries of absolute abundances of taxa were generated for all phyla, classes, orders, families, genera and species present in the dataset. The taxonomic summary tables were then rarefied to a depth of 10,000 counts per sample.

Shannon and Bray-Curtis indices were calculated with default parameters in R using the vegan library (Ihaka and Gentleman, 1996; Oksanen et al., 2007). The rarefied species data, taxonomic level 7, were used to calculated both indices. Plots were generated in R using the ggplot2 library (Hadley, 2016; Sievert et al., 2016). Significant difference among tested groups was determined using the Kruskal-Wallis one-way analysis of variance (ANOVA). The group significance tests were performed on the rarefied species data, taxonomic level 6 (genus), using the group_significance.py script within the QIIME v1.8 package.

The gene amplicon sequence data generated as part of this study have been submitted to the NCBI BioProject database under accession number (PRJNAXXXXX; to be determined).

### Behavior Testing

All behavior tests were conducted over a 2-week period with each test starting at the same time each morning. For each mouse, Elevated Plus Maze (EPM) was done first followed by Novel Object Recognition (NOR) the next day. Radial Arm Water Maze (RAWM) testing was then carried out starting the day after NOR.

### Elevated Plus Maze

A subset of young (*N* = 22) and old (*N* = 18) mice underwent three behavior tests.

The first test is the EPM. We used EPM to evaluate anxiety of the mice (Bachstetter et al., 2014; Parikh et al., 2016), which was also performed at Rodent Behavioral Core (RBC) at the UK. The EPM consists of two open and two closed arms elevated 100 cm above the floor. Closed arms are perceived as safe zones, and thus mice with higher anxiety had tendency to stay in the closed arms. We determined the anxiety-related behavior by measuring the time spent in the closed arms over the 5-min test session by EthoVision XT 8.0 video tracking software.

### Novel Object Recognition

The second behavioral test is the NOR, also performed in the RBC at the UK. NOR test is used to test spatial recognition memory (Lin et al., 2013). This task of recognition memory utilizes the fact that animals will spend more time exploring a novel object compared to an object that they are familiar with in order to satisfy their innate curiosity/exploratory instinct. Mice were given 15 min to explore two of the same objects in the “A/A” session. For the 10-min “A/B” test session, one of the A objects was replaced by a novel object (B). There was a 2-h delay between the A/A and A/B sessions. The total time mice spend investigating the objects was recorded and scored by the fully automated EthoVision XT 8.0 video tracking software. The D_2_ discrimination index was calculated by: D_2_ = (T_B_ − T_A_) ÷ (T_B_ + T_A_), where T_B_ is the time spent with the novel object B, and T_A_ is the time spent with the familiar object A.

### Radial Arm Water Maze

The third test is the RAWM, which is used to measure both spatial working memory and spatial reference memory (Arendash et al., 2001; Sood et al., 2007; Parikh et al., 2016). The RAWM task was conducted in the RBC at the UK as described previously (Guo et al., 2015), following a 2-day testing paradigm. A staggered training schedule was used, running the mice in cohorts of ten mice, while alternating the different cohorts through the trials over day 1 and day 2 of the test. This alternating protocol was used to avoid the learning limitations imposed by massed sequential trials and to avoid fatigue that may result from consecutive trials. Day 1 is the “learning” phase where mice went through three blocks (Blocks 1–3; 5 trials in each block) to test learning and short-term spatial memory. Day 2 is the “recall” phase where mice went through three additional blocks (Blocks 4–6) to test long-term memory after a 24-h retention period to locate the platform. It is expected that after the 2-day training, the mouse with intact memory can find the platform with minimal errors. Geometric extra-maze visual cues were fixed throughout the study on three sides of the curtains. Visual platform trials were included in the training and were used to determine if visual impairment could be a cofounding variable. Mouse performance was recorded by EthoVision XT 8.0 video tracking software (Noldus Information Technology) data analyzed by the EthoVision software for the number of incorrect arm entries, which are defined as errors. The video was reviewed for each mouse to ensure that the mice did not employ non-spatial strategies, such as chaining, to solve the task.

### Cerebral Blood Flow Measurement

A subset of young and old mice (*N* = 12 per group) were used to measure *in vivo* CBF using magnetic resonance imaging (MRI). MRI experiments were performed on a 7T MR scanner (Clinscan, Bruker BioSpin, Germany) at the MRI and Spectroscopy Center at the UK. Mice were anesthetized with 4.0% isoflurane for induction and then maintained in a 1.2% isoflurane and air mixture using a nose cone. Respiration rate (50–80 breaths/min) and rectal temperature (37 ± 1°C) were continuously monitored and maintained. T2-weighted structural images were acquired with field of view (FOV) = 18 × 18 mm^2^, matrix = 256 × 256; slice thickness = 1 mm, 10 slices, repetition time (TR) = 1500 ms and echo time (TE) = 35 ms. Quantitative CBF (with units of mL/g per minute) was measured using MRI-based pseudo-continuous arterial spin labeling (pCASL) techniques (Parikh et al., 2016). A whole body volume coil was used for transmission and a mouse brain surface coil was placed on top of the head for receiving. Paired control and label images were acquired in an interleaved fashion with a train of Hanning window-shaped radiofrequency pulses of duration/spacing = 200/200 μs, flip angle = 25° and slice-selective gradient = 9 mT/m, and a labeling duration = 2100 ms (Hong et al., 2015). The images were acquired by 2D multi-slice spin-echo echo planner imaging with FOV = 18 × 18 mm^2^, matrix = 128 × 128, slice thickness = 1 mm, 10 slices, TR = 4000 ms, TE = 35 ms and 120 repetitions. pCASL image analysis was employed with in-house written codes in MATLAB (MathWorks, Natick, MA, USA) to obtain quantitative CBF (Alsop et al., 2015).

### Blood-Brain Barrier Function Determination and Western Blotting

BBB function was determined by measuring P-glycoprotein (P-gp) transport activity in isolated brain capillaries. P-gp is an ATP-driven transporter highly expressed at the BBB that facilitates transport of Aβ from brain to blood. We previously established a confocal imaging-based assay to assess P-gp transport activity in freshly isolated brain capillaries from mice (Hartz et al., 2010, 2016). This assay measures within capillary lumens accumulation of [N-ε(4-nitro-benzofurazan-7-yl)-D-Lys(8)]-cyclosporin A (NBD-CSA), a fluorescent P-gp substrate.

#### Capillary Isolation

After euthanasia, mouse brain capillaries (*N* = 10 per group) were isolated according to a previously described protocol (Hartz et al., 2010, 2012). Briefly, mice were euthanized by CO_2_ inhalation and decapitated; brains were immediately harvested and collected in ice-cold DPBS buffer supplemented with 5 mM D-glucose and 1 mM Na-pyruvate, pH 7.4. Brains were dissected by removing meninges, choroid plexus and white matter, and homogenized in DPBS. The brain homogenate was mixed with Ficoll^®^ and centrifuged at 5800 *g* for 20 min at 4°C. The capillary pellet was resuspended in 1% BSA buffer and first passed through a 300 μm nylon mesh and then through a 27 μm nylon mesh. Capillaries retained by the 27 μm nylon mesh were collected and washed with DPBS buffer and used for experiments.

#### P-glycoprotein Transport Activity

Isolated capillaries were incubated for 1 h at room temperature with 2 μM NBD-CSA (custom-synthesized by R. Wenger (Basel, Switzerland)) in DPBS buffer. Ten capillary images were acquired by confocal microscopy (Leica TSP SP5 Confocal Microscope with Environmental Chamber, 63× D-Water UV objective, numerical aperture 1.2, Zoom: 4, 488-nm line of an argon laser, Leica Microsystems). Confocal images were analyzed by quantitating luminal NBD-CSA fluorescence with ImageJ software (v.1.45s; Wayne Rasband, NIH). Specific, luminal NBD-CSA fluorescence was taken as the difference between total luminal fluorescence and fluorescence in the presence of the P-gp-specific inhibitor PSC833 (5 μM, Novartis, Basel, Switzerland; Miller et al., 2008).

#### Western Blotting and Quantification

To determine protein expression, isolated brain capillaries were homogenized in tissue lysis buffer containing a cocktail of protease inhibitors. Homogenized brain capillary samples were centrifuged at 10,000 *g* for 15 min at 4°C, followed by a centrifugation of the denucleated supernatants at 100,000 *g* for 90 min at 4°C. Pellets (crude brain capillary plasma membranes) were resuspended and protein concentrations were determined using the Bradford protein assay. Normalized brain capillary membrane samples were separated on a NuPAGE™ 4%–12% Bis-Tris Protein Gels (1.0 mm, 15-wells; Thermo Fisher Scientific, Waltham, MA, USA) and transferred onto a Invitrolon™ PVDF membrane (0.45 μm pore size; Thermo Fisher Scientific, Waltham, MA, USA) membrane using the NuPAGE^®^ electrophoresis and blotting system (Invitrogen, Carlsbad, CA, USA). After protein transfer, the blotting membranes were incubated overnight with antibody to P-gp (C219; MA1-26528, ThermoFisher, 1 μg/ml) and β-actin (ab8226 from Abcam, 1:1000, 1 μg/ml). Proteins were detected using SuperSignal^®^ West Pico Chemoluminescent substrate (Pierce, Rockford, IL, USA) and protein bands were visualized with a BioRad Gel Doc™ XRS imaging system. P-gp was visualized first, membranes were then stripped with Restore™ Western Blot Stripping Buffer (Thermo Fisher Scientific, Waltham, MA, USA) and incubated with the antibody against β-actin. Image Lab 5.0 software from Bio-Rad Laboratories was used for densitometric analyses of band intensities and digital molecular weight analyses; the molecular weight marker was RPN800E (GE Healthcare, Chalfont St. Giles, Buckinghamshire, UK). Linear adjustments of contrast and brightness were applied to entire Western blot images. None of the Western blots shown were modified by nonlinear adjustments.

### Inducible Nitric-Oxide Synthase Measurement

Total RNA from frontal cortex and hypothalamus homogenate (*N* = 7–8 per group) was isolated using TRI Reagent solution (Ambion), and cDNA was synthesized from 1 μg total RNA from each individual sample using SuperScript III (Invitrogen). qRT-PCR was performed using TaqMan real time PCR (ViiA™7, Applied Biosystems). All reactions were performed with non-template negative control, and all data are mean ± SEM of two independent biological replicates. The gene probes and master mix were also purchased from Applied Biosystems. The probe sets were as follows: Mm00440502-m1 (iNOS Nos2), Mm00446968-m1 (hypoxanthine guanine phosphoribosyl transferase, Hprt). The relative expression levels were measured using the relative quantitation ΔCt (delta counts) method and normalized to Hprt.

### Metabolomic Profiling

A subset of mice was euthanized after 2- and 6-h of feeding (*N* = 6 and 4 for young and old mice, respectively) with subsequent collection of their whole brain. The brains were then sent to Metabolon (Durham, NC, USA) for analysis of brain metabolomic profile. Metabolon’s standard solvent extraction method was used to prepare the samples, which were then equally split for analysis via liquid chromatography/mass spectrometry (LC/MS) or gas chromatography/mass spectrometry (GC/MS) using their standard protocol (Evans et al., 2009).

#### Sample Preparation

Each sample was accessioned into a LIMS system, assigned a unique identifier, and stored at −70°C. To remove protein, dissociate small molecules bound to protein or trapped in the precipitated protein matrix, and to recover chemically diverse metabolites, proteins were precipitated with methanol, with vigorous shaking for 2 min (Glen Mills Genogrinder 2000) as described previously (Evans et al., 2009; Weiner et al., 2012). The resulting extract was divided into four fractions: one for analysis by ultra high performance liquid chromatography-tandem mass spectrometry run in positive mode (UPLC-MS/MS+), one for analysis by UPLC-MS/MS run in negative mode (UPLC-MS/MS−), one for analysis by GC-MS, and one aliquot was retained for backup analysis, if needed.

#### Mass Spectrometry Analysis

Non-targeted UPLC-MS/MS and GC-MS analyses were performed at Metabolon, Inc. as described (Evans et al., 2009; Sha et al., 2010; Weiner et al., 2012). The UPLC/MS/MS portion of the platform incorporates a Waters Acquity UPLC system and a Thermo-Finnegan LTQ mass spectrometer, including an electrospray ionization (ESI) source and linear ion-trap (LIT) mass analyzer. Aliquots of the vacuum-dried sample were reconstituted, one each in acidic or basic LC-compatible solvents containing eight or more injection standards at fixed concentrations (to both ensure injection and chromatographic consistency). Extracts were loaded onto columns (Waters UPLC BEH C18-2.1 × 100 mm, 1.7 μm) and gradient-eluted with water and 95% methanol containing 0.1% formic acid (acidic extracts) or 6.5 mM ammonium bicarbonate (basic extracts). The instrument was set to scan 99–1000 m/z and alternated between MS and MS/MS scans.

Samples destined for analysis by GC-MS were dried under vacuum desiccation for a minimum of 18 h prior to being derivatized using bis(trimethylsilyl)trifluoroacetamide (BSTFA) as described (Ohta et al., 2009). Derivatized samples were separated on a 5% phenyldimethyl silicone column with helium as carrier gas and a temperature ramp from 60°C to 340°C within a 17-min period. All samples were analyzed on a Thermo-Finnigan Trace DSQ fast-scanning single-quadrupole MS operated at unit mass resolving power with electron impact ionization and a 50–750 atomic mass unit scan range. The instrument is tuned and calibrated for mass resolution and mass accuracy daily.

#### Quality Control

All columns and reagents were purchased in bulk from a single lot to complete all related experiments. For monitoring of data quality and process variation, multiple replicates of extracts from a pool of human plasma were prepared in parallel and injected throughout the run, interspersed among the experimental samples. Instrument variability was determined by calculating the median relative standard deviation (RSD) for the standards that were added to each sample prior to injection into the mass spectrometers (median RSD = 4%; *n* = 21 standards). Overall process variability was determined by calculating the median RSD for all endogenous metabolites (i.e., non-instrument standards) present in 100% of technical replicate samples created from a homogeneous pool containing a small amount of all study samples (median RSD = 6%; *n* = 170 metabolites). In addition, process blanks and other quality control samples are spaced evenly among the injections for each day, and all experimental samples are randomly distributed throughout each day’s run.

#### Compound Identification, Quantification and Data Curation

Metabolites were identified by automated comparison of the ion features in the experimental samples to a reference library of chemical standard entries that included retention time, molecular weight (m/z), preferred adducts and in-source fragments as well as associated MS spectra and curated by visual inspection for quality control using software developed at Metabolon (Dehaven et al., 2010). Identification of known chemical entities was based on comparison to metabolomic library entries of more than 2800 commercially-available purified standards. Subsequent QC and curation processes were utilized to ensure accurate, consistent identification and to minimize system artifacts, mis-assignments and background noise. Library matches for each compound were verified for each sample. Peaks were quantified using area under the curve. Raw area counts for each metabolite in each sample were normalized to correct for variation resulting from instrument inter-day tuning differences by the median value for each run-day, therefore setting the medians to 1.0 for each run. This preserved variation between samples, but allowed metabolites of widely different raw peak areas to be compared on a similar graphical scale. Missing values were imputed with the observed minimum after normalization.

#### Bioinformatics

The LIMS system encompasses sample accessioning, preparation, instrument analysis and reporting and advanced data analysis. Additional informatics components include data extraction into a relational database and peak-identification software; proprietary data processing tools for QC and compound identification; and a collection of interpretation and visualization tools for use by data analysts. The hardware and software systems are built on a web-service platform utilizing Microsoft’s .NET technologies which run on high-performance application servers and fiber-channel storage arrays in clusters to provide active failover and load-balancing.

### Statistical Analysis

All statistical analyses were completed using GraphPad Prism (GraphPad, San Diego, CA, USA). One-tailed Student’s *t*-test was performed for determination of differences between groups. Levels of statistical significance were reached when *p* < 0.05. For Metabolon, missing values in the data are assumed to be below the level of detection of the used instruments. Log transformations and imputation of missing values with the minimum observed values for each metabolite was conducted. This was followed by ANOVA to identify biochemicals that were significantly different between groups. Given the multiple comparisons inherent in analysis of metabolites, between-group relative differences are assessed using both *p*-value and false discovery rate analysis (q-value).

## Results

### Altered Gut Microbiome and Increased Body Weight with Age

**Figure 1:**
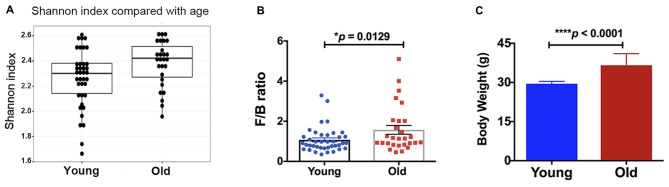
**(A)** The old mice showed a higher alpha-diversity, as indicated by the Shannon index, than the young mice (*p* = 0.02). Compared with the young mice, the old mice had significantly increased **(B)** *Firmicutes/Bacteroidetes* ratio and **(C)** body weight. *N* = 39 and 28 for young and old mice, respectively. Data are presented as mean ± SEM.

Alpha diversity (e.g., Shannon index, H value) was measured for fecal microbial communities of all samples, at the taxonomic level of genus (Figure 1A). Older mice had a significantly higher alpha diversity than young mice (Kruskal-Wallis *p*-value = 0.022). Although no significant differences in beta diversity were observed (analysis of similarity, ANOSIM R statistic = 0.006, *p*-value = 0.307, 999 permutations), and no specific taxa were significantly different between the groups of young and old mice (Kruskal-Wallis, false discovery rate, FDR-corrected *p*-value < 0.05), the ratio of *Firmicutes/Bacteroidetes* was significantly different between age groups. Old mice had significantly higher *Firmicutes*/*Bacteroidetes* (F/B) ratio (Figure 1B, 46% increase, *p* < 0.05). Further, we found that the body weight of the old mice was significantly higher compared with that of the young mice (Figure 1C, 24% increase, *p* < 0.05).

### Enhanced Proinflammatory Metabolism with Age

**Figure 2:**
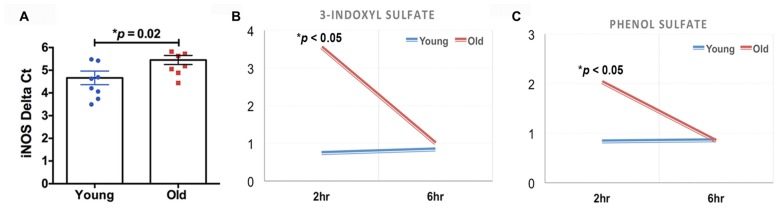
**(A)** The old mice had a significant increase in inducible nitric oxide synthase (iNOS) in the brain compared to the young mice (*N* = 7–8 per group). The old mice had acute elevations (2 h after feeding) in **(B)** 3-indoxyl sulfate and **(C)** phenol sulfate; those levels returned to baseline 4 h later (*N* = 6 and 4 for young and old mice, respectively).

The quantification of the brain metabolites is shown in Table 1. We observed several significant age-dependent changes in basal brain metabolites (after 6 h of final feeding). Notably, the old group had a 31%–83% change in markers of the methionine cycle with significantly greater methionine, cysteine, cysteine-glutathione disulfide and cystathionine. First, methionine is an amino acid that may be accountable for increased mitochondrial reactive oxygen species (ROS; Pamplona and Barja, 2006). Due to this, we believe the old mice exhibit increased oxidative stress in the brain. We also found lipids related to inflammatory responses significantly elevated in the old mice, including a 82% increase in prostaglandin D2 and a 80% increase in prostaglandin E2. Further, markers associated with AD were significantly greater in the old mice compared to the young, including a 21% increase in 24(S)-hydroxycholesterol, 28% increase in mead acid (20:3n9), 37% increase in phenylalanine, 58% increase in spermidine, 22% increase in docosapentaenoate (n6 DPA; 22:5n6), 11% increase in creatine and 12% increase in phosphocholine (Morrison and Kish, 1995; Geddes et al., 1997; Lütjohann et al., 2000; García-Calatayud et al., 2005; Gallant et al., 2006; Astarita et al., 2011; Wissmann et al., 2013). In contrast, CDP-choline, a metabolite that has shown to alleviate AD symptoms (Alvarez et al., 1999), saw a 6% decrease in the old group. In addition, old mice also demonstrated a 114% increase in citrate. Collectively, the metabolic profiling indicates that the old mice had enhanced proinflammatory metabolism. These data are consistent with our observation of significantly increased iNOS levels in the brain of old mice compared to that of young mice (Figure 2A, 17% increase, *p* = 0.02).

**Table 1:** The metabolomics profiling of brain metabolites is drastically modified with age.

Classification	Metabolite	*P*	Young	Old
Amino acids	Creatine	0.003	0.990 ± 0.022	1.101 ± 0.017
	Cystathionine	0.026	0.943 ± 0.072	1.316 ± 0.174
	Cysteine	0.007	1.023 ± 0.138	1.873 ± 0.262
	Cysteine-glutathione disulfide	0.013	1.008 ± 0.132	1.736 ± 0.267
	Methionine	0.001	0.915 ± 0.047	1.204 ± 0.015
	Phenylalanine	0.002	0.917 ± 0.042	1.255 ± 0.085
	Spermidine	0.016	0.910 ± 0.059	1.436 ± 0.238
Cofactors and vitamins lipids	Citrate	0.008	0.867 ± 0.099	1.860 ± 0.381
	24(S)-hydroxycholesterol	0.025	0.939 ± 0.027	1.135 ± 0.098
	CDP-choline	0.037	1.002 ± 0.014	0.944 ± 0.028
	Docosapentaenoate (n6 DPA; 22:5n6)	0.007	0.994 ± 0.019	1.215 ± 0.085
	Mead acid (20:3n9)	0.025	1.070 ± 0.068	1.367 ± 0.122
	Phosphocholine	0.024	1.076 ± 0.029	1.206 ± 0.054
	Prostaglandin D2	0.009	0.682 ± 0.087	1.238 ± 0.193
	Prostaglandin E2	0.007	0.746 ± 0.076	1.341 ± 0.207

*Representative data, shown in scaled intensity, of the young and old groups. Data are mean ± SEM*.

We found that two of the brain metabolites related to gut microbiota were also significantly different between young and old cohorts. Compared to the young mice, old mice had significantly higher 3-indoxyl sulfate (Figure 2B, 380% increase, *p* < 0.05) and phenol sulfate (Figure 2C, 144% increase, *p* < 0.05) in their brain within 2 h after their final feeding, however, the levels of the two metabolites returned to the baseline after 6 h of feeding. 3-indoxyl sulfate is generated in the liver as a result of gut microbiome metabolism of tryptophan; phenol sulfate is a metabolite derived from bacterial metabolism of phenylalanine. The results suggest that the gut microbiome may play an important role in modulating brain metabolism. Because 3-indoxyl sulfate and phenol sulfate both are related to neurological toxicity and inflammation (Zgoda-Pols et al., 2011; Weber et al., 2015), it implies that old mice had higher neuroinflammation, which may of resulted from age-dependent changes in the gut microbiota.

### Impaired Neurovascular Functions with Age

**Figure 3:**
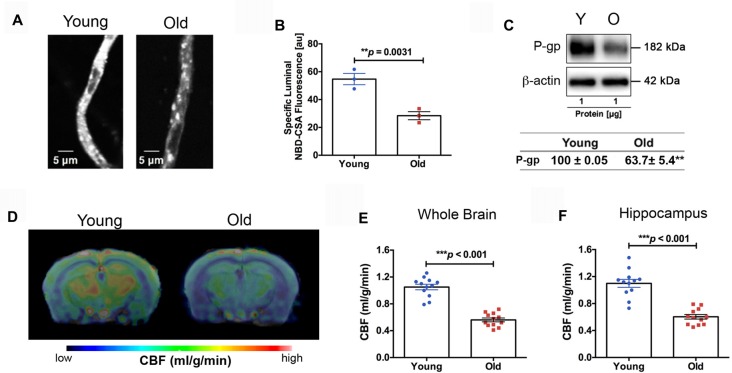
**(A)** Representative confocal images showing decreased luminal accumulation of N-ε(4-nitro-benzofurazan-7-yl)-D-Lys(8)-cyclosporin A (NBD-CSA) fluorescence (white) in brain capillaries isolated from the old mice compared to young mice, indicating reduced P-glycoprotein (P-gp) activity. **(B)** Corresponding quantitative fluorescence data; images are shown in arbitrary fluorescence units (scale 0–255). Data are mean ± SEM for 10 capillaries from one preparation of 10 mice. **(C)** Western blotting (WB) for P-gp from the cortical vasculature, β-Actin was used as loading control (top); corresponding values are shown in the table (bottom). The WB data from the old mice were normalized to β-Actin and compared to the young mice (100%), **p < 0.01. **(D)** Cerebral blood flow (CBF) maps superimposed on structural images; the color code indicates the level of CBF on a linear scale. Quantitative CBF (ml/g/min) obtained from **(E)** the whole brain and **(F)** hippocampus (*N* = 12 per group). Data are mean ± SEM.

We found impaired BBB function in the old mice. Figure 3A shows representative confocal images of capillaries; the intensity of fluorescence in the capillary lumen reflects the amount of P-gp, an efflux transporter of Aβ. The corresponding quantitative results are shown in Figure 3B; the old mice had significantly reduced P-gp transport activity (*p* = 0.0031) compared to the young mice. We also measured P-gp protein expression levels (Figure 3C). Similar to the results of P-gp activity, we found that the old mice had significant reduction in P-gp protein levels compared to the young mice (decrease to 63.7 ± 5.4% over 100% young; *p* < 0.001; Figure 3C). We also observed reduced CBF in the old mice. Figure 3D shows the representative CBF images of the young and old mice. The CBF level is colorized in a linear scale, indicating that the young mice have overall higher CBF compared to the old mice, which was confirmed by the quantitative global CBF values (Figure 3E, 87% increase, *p* < 0.001). We did further CBF analyses in brain regions associated with cognitive functions (e.g., memory and learning) based on MRI structural imaging and mouse brain atlas. We found that young mice exhibited an 82% increase in CBF in the hippocampus (*p* < 0.001, Figure 3F), compared to the old mice.

### Compromised Cognition and Increased Anxiety with Age

**Figure 4:**
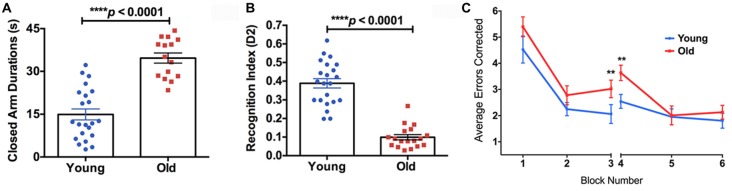
**(A)** The elevated plus maze (EPM) found the old mice to have a significantly higher closed arm duration compared to the young group. **(B)** The Novel Object Recognition (NOR) test found the old group had a significantly lower recognition index, or D_2_, than the young group. **(C)** The average errors made by the young and old mice during the Radial Arm Water Maze (RAWM) split into six blocks. The significant difference between the two groups in average errors corrected showing in Block 3 (*p* = 0.0307) and Block 4 (*p* = 0.0045). *N* = 22 and 18 for young and old mice, respectively. Data are presented as mean ± SEM. **p < 0.05.

The old mice spent significantly longer time in the closed arms compared to the young mice in the EPM test (Figure 4A, 132% increase, *p* < 0.0001), indicating higher anxiety. In the NOR test, the old mice showed a significantly lower D_2_ value compared to the young group, suggesting reduced recognition memory (Figure 4B, −74% decrease, *p* < 0.0001). In the RAWM test, the old group made significantly more errors in the learning phase (Block 3; 46% increase, *p* < 0.01) and the initial recall phase (Block 4; 43% increase, *p* < 0.01), compared to the young group (Figure 4C).

## Discussion

In this study, we demonstrated the age-dependent changes in brain metabolism, gut microbiome, vascular functions, memory and anxiety. Specifically, aged mice had enhanced proinflammatory, increased ratio of *Firmicutes* to *Bacteroidetes*, increased bacterial alpha diversity and body weight, impaired BBB and CBF, compromised learning and long-term memory, and increased anxiety. These deleterious changes in aging have the potential to increase the risk for neurological disorders and dementia, including AD.

We found several amino acid increased in the old mice that are highly associated with AD, including spermidine, phenylalanine, creatine citrate and methionine-related metabolites. First, spermidine levels have been found to be higher in the temporal cortex with a trending elevation in hippocampus and frontal cortex in AD patients, potentially as a response to brain injury (Morrison and Kish, 1995). Excess spermidine could exacerbate neurodegeneration as it positively modulates N-methyl-D-aspartate (NMDA) glutamate receptor function and disrupt calcium homeostasis. Second, high phenylalanine levels are also found in AD patients, associated with immune activation (Wissmann et al., 2013). Phenylalanine disturbs neopterin and tryptophan metabolism, which is correlated with cognitive impairment (Keegan et al., 2016; Palermo et al., 2017). Third, old mice had higher creatine deposits in the brain, consistent with literature that creatine metabolism malfunction plays an important role in AD (Gallant et al., 2006). Fourth, old mice had increased levels of citrate in the brain suggesting increased usage of fatty acid synthesis for membrane remodeling associated with aging due to the increased stress and inflammation that damage neuronal membranes (Curi et al., 2017). Finally, the old mice had altered methionine-associated metabolism, including increased methionine, cystathionine, cysteine and cysteine-glutathione disulfide. Excessive methionine has been considered to be accountable for increased mitochondrial ROS production, which in turn enhances oxidative stress and inflammation (Jaeschke, 2011; Schweinberger and Wyse, 2016; Palermo et al., 2017). Interestingly, previous studies have shown that methionine restriction can lead to increased longevity by decreasing mitochondrial complex IV activity and accumulation of ROS (Kozieł et al., 2014; Brown-Borg, 2016).

Accumulation of lipids in the brain is another hallmark of AD (Alzheimer et al., 1995). In the present study, we also found AD-associated fatty acids increased in the aged mice. First, 24(S)-hydroxycholesterol, an oxidized product of cholesterol produced in the brain, has been demonstrated to be elevated in AD patients and is hypothesized to be an early marker for distorted cholesterol status (Lütjohann et al., 2000). Second, phosphocholine has been revealed to be increased in rats during the early stages of AD during lesion-induced neuronal sprouting in the hippocampus (Geddes et al., 1997). This indicates that sprouting may occur early on in AD with phosphocholine as a marker. In addition, sprouting may also lead to a decline in energy metabolism due to energy being used for sprouting, consistent with the evidence of glucose metabolism decline in AD. Third, mead acid (20:3n9), an omega-9 fatty acid, has been demonstrated to be increased in the mid-frontal cortex, temporal cortex and hippocampus of AD patients (Astarita et al., 2011). Fourth, docosapentaenoate (n6 DPA), an omega-6 fatty acid, has been inversely correlated with learning (García-Calatayud et al., 2005). This is consistent with previous findings that oleic acid-enriched triglycerides were found in the brain of a triple transgenic mice model of AD (3xTg-AD); but when the triglycerides were inhibited, proper brain function was restored (Hamilton et al., 2015). Further, prostaglandin D2 and E2, produced from arachidonate, are generators of an inflammatory response and present in increased amounts in such an event, were also elevated in the old group (Ricciotti and Fitzgerald, 2011). Specifically in one study, prostaglandin E2 was associated with neuronal oxidative damage after activation by lipopolysaccharide (LPS; Montine et al., 2002). LPS also can activate iNOS, which is consistent with our finding that old mice had significantly increased iNOS expression in the brain. In contrast, the aged mice showed reduced levels of CDP-choline, which has shown to alleviate AD symptoms by increasing CBF and brain electrical activity, and reducing serum cytokine IL-1β levels (Alvarez et al., 1999). Old mice also had dramatically decreased levels of glycerophopshorylcholine (GPC), which has been used to treat patients with cognitive impairment and AD (Parnetti et al., 2007). Collectively, old mice exhibited a myriad of markers associated with inflammation and AD.

The enhanced inflammation with age may have also occurred in the gut. We found that 3-indoxyl sulfate and phenol sulfate, which originate from the gut microbiota, were significantly elevated in the aged mice’s brains after meals. Significant increases of indoxyl sulfate have shown to enhance neurological toxicity, increase oxidative stress and ROS, and induce endothelial dysfunction by inhibiting endothelial proliferation and migration *in vitro* (Brunet et al., 2003; Dou et al., 2007). In addition, phenol sulfate is derived from bacterial metabolism of phenylalanine, a marker for AD, as mentioned above. Next, we found a modest increase in older mice in the relative abundance of sequences from bacteria of the phylum *Firmicutes*, with a concomitant decrease in the relative abundance of sequences from bacteria of the phylum *Bacteroidetes*. Although neither change was statistically significant, the overall change, leading to an increased *Firmicutes/Bacteroidetes* (F/B) ratio in older mice, was significant. As bacteria from the *Firmicutes* have been associated with weight-gain and those within the *Bacteroidetes* with weight-loss, this increased F/B ratio may promote body weight gain in old mice (Turnbaugh et al., 2006). Further, this increased F/B ratio may lead to excessive low-grade inflammation and a substantial increase in energy harvesting and food intake (Cani et al., 2012; Harris et al., 2012). As a result, the F/B ratio has become a well-known marker for obesity and Type 2 Diabetes Mellitus (T2DM; Walters et al., 2014). Interestingly, AD has been called Type 3 diabetes due to the overlapping symptoms observed in T2DM, e.g., insulin resistance and increased inflammation in the brain (de la Monte and Wands, 2008), along with an increased risk of dementia (Vagelatos and Eslick, 2013). Thus, the increased F/B ratio may be indicative of an increased risk for AD.

Short-chain fatty acids (SCFAs), including butyrate and propionate that are produced by certain bacterial species, have a dramatic impact on brain function. For example, butyrate has been demonstrated to prevent inflammatory responses via NFκB inhibition in microglia and hippocampal slice cultures (Huuskonen et al., 2004). Also, indole-3-proprionic acid (IPA), another metabolite produced by the gut microbiome, was demonstrated to inhibit Aβ fibril formation in neurons and neuroblastoma cells (Chyan et al., 1999). On the contrary, increased *Firmicutes* may enhance trimethylamine (TMA) and its co-metabolites trimethylamine N-oxide (TMAO; Martínez-del Campo et al., 2015). TMAO level predicts risk for atherosclerosis and directly induces cardiovascular disease (Wang et al., 2011, 2014; Bennett et al., 2013; Zhang and Davies, 2016).

Reduced SCFAs has been shown to induce BBB permeability (Braniste et al., 2014). This is consistent with our findings that BBB function was compromised in the old mice. Specifically, we found the old mice had a significantly lower quantity of P-gp transporters in BBB, which are responsible for Aβ clearance from the brain to blood, indicative of an increased risk of developing AD (Cirrito et al., 2005). BBB breakdown has been caused by elevated neuroinflammation (Bell et al., 2012) and is associated with CBF reduction. In line with this, we found decreased CBF in old mice. In particular, old mice had significantly decreased CBF in the hippocampus, the brain region that modulates cognitive function. Further, the old mice performed worse in the RAWM and NOR behavior tests than the young animals. Specifically, the old group performed worse in Block 3 and 4 of the RAWM compared to the young group, exhibiting inhibited spatial learning and long-term memory formation, consistent with our previous findings (Guo et al., 2015; Parikh et al., 2016). In the NOR test, the old group had a worse D_2_ score compared to the young group, indicating a worse recognition memory. Taken together, our findings are consistent with the literature where impaired neurovascular integrity plays a critical role in determining cognitive functions (Birdsill et al., 2013).

Higher anxiety levels were shown in old mice compared to young mice, which may be also associated with brain vascular and metabolic dysfunctions (Gur et al., 1987; Ebmeier et al., 1997; Park and Moghaddam, 2017). Interestingly, the gut microbiome has been linked with anxiety (Foster and McVey Neufeld, 2013). Indeed, the gut and brain are connected via the enteric nervous system and have bi-directional communication, impacting behavior (Schnorr and Bachner, 2016). Certain bacterial species such as *L. rhamnosus* have been demonstrated to decrease anxiety like symptoms and stress induced hormones (Bravo et al., 2011). Studies using oral administration of food-borne pathogens showed evidence that bacteria residing in the gastrointestinal tract can activate stress circuits through activation of vagal pathways (Lyte et al., 2006; Goehler et al., 2008). Exposure to a subpathogenic infection of *C. jejuni* increased anxiety-like behavioral measure in the EPM 2 days after infection (Lyte et al., 1998). These studies clearly demonstrated that inflammatory state could have strong influences on behavior and mental health. Conversely, treating mice with probiotics has been shown to reduce anxiety-like behavior; the probiotic-treated group showed increased entries into the open arms of the EPM compared to the control group (Casey et al., 2011). Further confirming the importance of the gut microbiome in relation to stress, one study demonstrated germ-free mice to have an excessive release of stress hormones. However, when these mice were colonized with *Bifidobacterium infantis*, this response was alleviated (Sudo et al., 2004). Similar observations were also made in clinical trials in patients with chronic fatigue, showing that anxiety-like symptoms were alleviated by probiotics (Rao et al., 2009). The study findings may be applicable to AD as increased anxiety levels and depression are commonly found preceding the onset of AD (Ferretti et al., 2001). Modulating the gut microbiome may thus be important for reducing risk or preventing AD and other neurodegenerative disorders (Hill et al., 2014; Hu et al., 2016; Fung et al., 2017; Tremlett et al., 2017).

**Figure 5:**
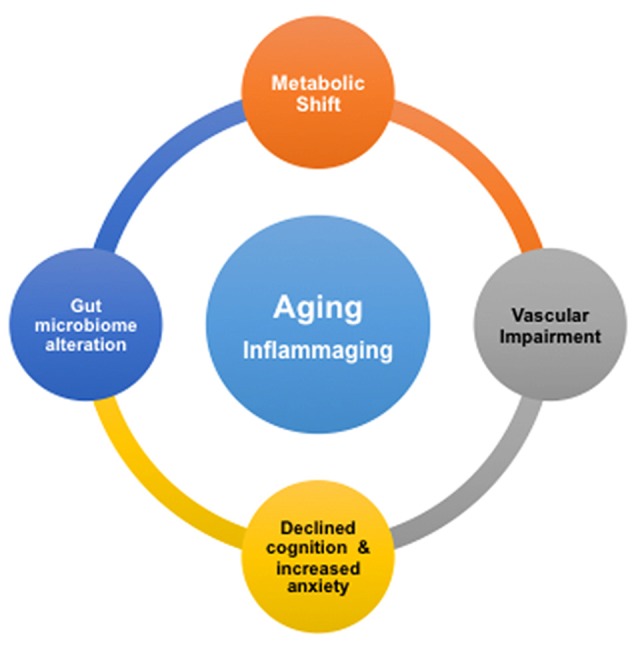
Proposed associations of age-dependent changes in brain metabolism, vascular integrity, gut microbiome, cognition and anxiety level. Inflammation in aging, or inflammaging, might play a critical role in be a driving the deleterious changes in the brain and gut.

We summarize our findings with Figure 5. It shows that advancing age drives deleterious modifications in metabolism, gut microbiome, neurovascular integrity, cognition and mental health, which may significantly enhance the risk for AD. Our study implies that inflammation may play a critical role in the remodeling process. This is consistent with the concept of inflammaging, the phenomenon where innate immunity is activated, coupled with the rise of proinflammation with advancing age (Xia et al., 2016). We suggest that, based on our results, the inflammatory responses with age are systematic; they may be originated from the CNS as well as the peripheral systems, e.g., from the gut. To promote healthy aging and prevent AD, it will thus be critical to manage low-grade, chronic proinflammation over time.

In future studies, it is important to determine mechanisms linking the brain and gut in the context of brain aging, including pathways involved in SCFAs, neurotransmitters, vagus nerve activity and immune system function. As well as, to identify potential nutritional interventions that can promote brain-gut interactions, such as probiotics and prebiotics.

We have recently shown that dietary interventions can delay brain aging (e.g., caloric restriction and rapamycin) and thus, it will be crucial in the future to determine if these dietary interventions also have a significant impact on the brain-gut axis (Guo et al., 2015; Parikh et al., 2016; Lin et al., 2017). In addition, it will be imperative to develop surrogate biomarkers using neuroimaging. In the present study, we used MRI to measure *in vivo* CBF but we have also developed imaging methods to determine brain metabolic and anatomical integrity (Lin et al., 2014, 2015). Further, we will also use this state-of-the-art technology to study brain-gut axis and make our research strategy translatable to clinical applications.

In conclusion, we found the inflammation-associated impact on brain metabolism, gut microbiome, neurovascular functions, memory and anxiety in aging mice. However, additional research needs to be conducted on the gut microbiome and mechanisms of the gut-brain axis. Understanding brain aging is imperative to identify risks, and intervention thereof, for AD. A comprehensive and integrative characterization of brain aging, including its crosstalk with peripheral systems and factors, will help to define the mechanisms underlying the shift from normal aging to pathological processes in the etiology of AD (Hill et al., 2014; Fung et al., 2017).

## Author Contributions

JDH, IP and A-LL contributed to the major design, acquisition, analysis and interpretation of data for the work. SJG, GC, IP and JDH contributed to the gut microbiome analysis. AMSH and BB contributed to data acquisition and analysis related to BBB functions. RPM and MK contributed to biostatistical support for the metabolomic profiling. JDH, IP, SJG, GC, RPM, AMSH, BB and A-LL drafted and revised the work for important intellectual content, approved of the final version and agreed to be accountable for all aspects of the work in ensuring that questions related to the accuracy or integrity of any part of the work are appropriately investigated and resolved.

## Conflict of Interest Statement

The authors declare that the research was conducted in the absence of any commercial or financial relationships that could be construed as a potential conflict of interest.
